# Phenotypic similarity of adverse drug reactions and disease phenotypes is a bridge to mechanistic discovery

**DOI:** 10.1038/s44386-025-00021-6

**Published:** 2025-08-04

**Authors:** Farzaneh Firoozbakht, Nina Wenke, Olga Tsoy, Joseph Loscalzo, Jan Baumbach, Maria Louise Elkjaer

**Affiliations:** 1 Institute for Computational Systems Biology, University of Hamburg, Hamburg, Germany; 2 Department of Medicine, Brigham and Women’s Hospital, Harvard Medical School, Boston, MA USA; 3 Department of Mathematics and Computer Science, University of Southern Denmark, Odense, Denmark

**Keywords:** Drug safety, Pharmacology

## Abstract

Adverse drug reactions (ADRs) remain a major barrier to safe therapeutic developments. A key challenge is our limited understanding of their underlying mechanisms. In this study, we investigated whether ADRs and diseases phenotypes (DPs) with similar clinical manifestations share mechanistic similarities. To this end, we constructed a comprehensive knowledge graph and applied a graph representation learning to quantify mechanistic similarities between phenotypically similar ADRs and DPs. Our analysis reveals substantial mechanistic overlap among ADRs and DPs within specific system organ classes, including cardiac, psychiatric, and metabolic disorders. These findings suggest that drugs interacting with proteins linked with specific DPs are more likely to cause ADRs with similar phenotypes. By integrating drug-induced and disease-related phenotypes, our approach offers new insights into ADR mechanisms and supports the prioritization of drugs with lower ADR risk. This work contributes to advancing safer and more targeted therapeutic development by bridging phenotypic similarity and molecular mechanisms.

## Introduction

Adverse drug reactions (ADRs) cause significant challenges in clinical practice and drug development. Even after a drug is marketed, its safety profile in clinical use often remains incomplete as unintended side effects or ADRs may emerge beyond the drug’s intended therapeutic effects^1,2^. Some ADRs can be severe or even life-threatening. For example, thalidomide, initially marketed as an anti-nausea medication for pregnant women, was later found to cause limb deformities (phocomelia) in newborns^3,4^. Similarly, fialuridine, used for hepatitis B virus infection during phase II clinical trials, led to fatal liver and kidney failure in five out of 15 patients and required emergency liver transplantation in two others^5^. These examples underscore the importance of predicting the secondary mechanisms of drugs that could lead to ADRs prior to clinical use to ensure patient safety and mitigate harmful outcomes.

ADRs are commonly classified into two types: Type A (pharmacological) and Type B (idiosyncratic). Type A ADRs, which account for ~75% of all ADRs, are dose-dependent and typically predictable based on the drug’s known pharmacological properties, particularly its interactions with biological targets. In contrast, Type B ADRs are not dose-dependent and are unpredictable, as they cannot be explained by the drug’s established pharmacology. Hence, the mechanisms of many ADRs can be attributed to the off-target effects of the drugs that induce them. However, drugs often interact with multiple protein targets, leading to diverse downstream effects. To narrow down these effects to more biologically relevant protein sets, additional sources of information, such as human genetic data, can be integrated^6,7^. For example, in our previous study^6^, we developed a pipeline, called DREAMER, that integrates human genetic data with drug target information to identify the proteins associated with clinical phenotypes.

Certain ADRs share the same clinical phenotype as DPs^8^ raising the question of whether such phenotypic similarities at a certain level of medical nosology also reflect shared molecular mechanisms? For example, the mechanism underlying ventricular tachycardia as an ADR is well established, often involving hERG channel inhibition, which increases susceptibility to torsade de pointes, a form of polymorphic ventricular tachycardia. These known mechanisms suggest that ADRs and DPs with similar clinical phenotypes may share molecular pathways. Building on such examples, this study explores whether similar relationships extend to other ADR–DP pairs with less well-understood mechanisms. Addressing this question provides a dual perspective that deepens our understanding of shared ADR–DP mechanisms and provides a more robust foundation for identifying these biological pathways.

In this study, we specifically investigate whether phenotypic similarity between ADRs and DPs can serve as a bridge to uncover shared molecular mechanisms. Moreover, we examine whether this relationship holds universally across all clinical phenotypes or is confined to specific cases. To address these questions, we constructed a comprehensive *knowledge graph* (KG) integrating drugs, diseases, ADRs, DPs, and proteins as nodes. To simplify complex interactions within the KG, we employed graph representation learning, i.e., methods that encode nodes into low-dimensional vector representations while preserving the structural and relational information of the graph. These methods enable us to quantify mechanistic similarities between phenotypically similar ADRs and DPs. By analyzing the similarities of both drug-induced and disease-related phenotypes, our study provides a framework for gaining deeper insights into the molecular mechanisms underlying clinical phenotypes and early identification of ADRs. In particular, our results suggest that drugs targeting proteins in proximity to those associated with specific DPs tend to exhibit ADRs with clinical phenotypes similar to the given DP. This insight aids in prioritizing drugs less likely to cause ADRs, enhancing drug safety and efficacy^9^. Throughout this manuscript, we use the term “phenotype” to refer specifically to clinical phenotype.

## Results

### Knowledge graph construction

**Fig. 1:**
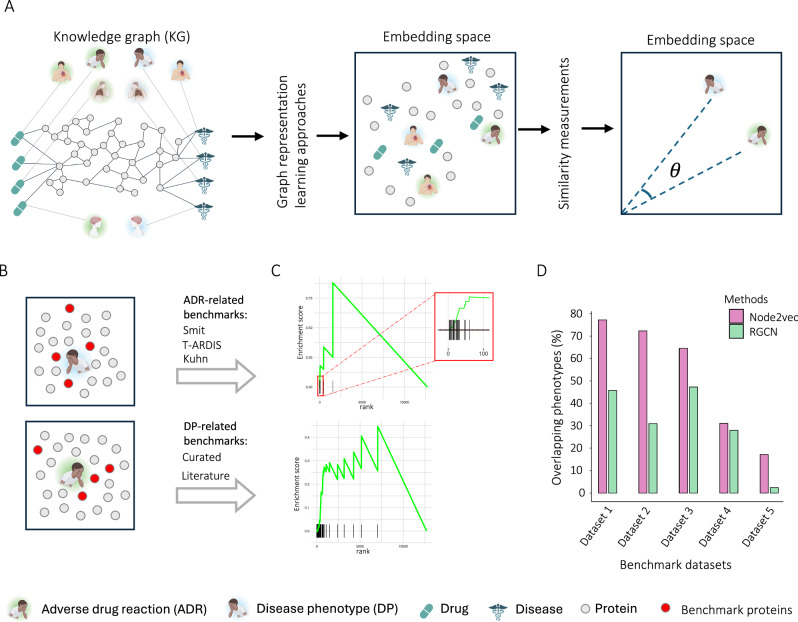
The overview of our analysis pipeline and biological evaluation of the embedding space. **A** schematic representation of our pipeline including knowledge graph (KG) construction, representing the KG into an embedding space, and using cosine similarity to measure network proximities between nodes. **B** Visualization of proteins in close proximity to ADR (blue shaded–upper plot) and DP (green shaded–lower plot). Red points indicate proteins reported by the benchmark datasets for a specific ADR or DP. **C** Statistical evaluation using gene set enrichment analysis (GSEA) to assess the significance of benchmark proteins in the neighborhoods of ADRs and DPs. **D** Percentage of phenotypes with significant enrichment of benchmark proteins in their neighborhoods (adjusted *p* < 0.05). The results highlight the superior performance of node2vec in capturing protein-phenotype relationships compared to relational graph convolutional networks (RGCN). Benchmark proteins are obtained from Smit (Dataset 1), T-ARDIS (Dataset 2), Curated (Dataset 3), literature-based associations for DP–proteins (Dataset 4), and Literature-based associations for ADR–proteins (Dataset 5)—Methods. Created in BioRender. Baumbach, J. (2025) https://BioRender.com/6otv8kf.

We integrated data from several databases^8,10–14^ to investigate whether there is any common mechanism between ADRs and DPs that exhibit the same phenotypes. The mechanisms underlying many phenotypes can be explained through proteins associated with them. Specifically, ADR mechanisms are often linked to the off-target effects of drugs, while DP mechanisms are associated with proteins (or genes) associated with diseases. Since no large and experimentally validated databases directly link ADRs (or DPs) to proteins, we leveraged known drug–protein and disease–gene associations by using drugs (for ADRs) and diseases (for DPs) as intermediary nodes (Fig. 1A). This approach enabled network-based inference to establish indirect connections between phenotypes and proteins and investigate the shared mechanisms of ADRs and DPs exhibiting the same phenotype.

Accordingly, we constructed a KG where nodes represent drugs, diseases, proteins, ADRs, and DPs. Network edges include ADR–drug, drug–protein, disease–gene, gene–protein, disease–DP, and protein–protein interaction (PPI) to capture the interconnected nature of protein operations within cells (Fig. 1A). To focus on biologically relevant relationships, we restricted our KG to pairs of ADRs and DPs recognized as phenotypically equivalent based on the ADR–DP relationships deposited in the *BioPortal* database^8^. Summary statistics and data sources of our KG are provided in Tables 1 and 2, with a detailed description available in our previous study^6^.

**Table 1:** The summary statistics of nodes in our knowledge graph

Node type	Number of nodes	Node ID	Resource links
Adverse drug reaction	619	MedDRA^15^	https://www.meddra.org/
Disease phenotype	619	HPO^11^	https://hpo.jax.org/
Drug	409	Drugbank^12^	https://go.drugbank.com/
Disease	2518	Mondo Disease Ontology^52^	https://mondo.monarchinitiative.org/
Gene	3245	DisGeNET^13^	https://disgenet.com/
Protein	12694	UniProt^53^	https://www.uniprot.org/

**Table 2:** The summary statistics of edges in our knowledge graph

Edge type	Database	Number of edges	Resource links
ADR–[is phenotypically similar to]–DP	BioPortal^8^	619	https://bioportal.bioontology.org/ontologies/HP
ADR–[is reported for]–Drug	Sider^10^	5629	http://sideeffects.embl.de/
Drug–[has target]–Protein	DrugBank and DrugCentral^12,50^	3741	https://go.drugbank.com/
Phenotype–[is reported for]–Disease	HPO^11^	9186	https://hpo.jax.org/
Gene–[associated with]–Disease	DisGeNET^13^	6479	https://disgenet.com/
Gene–[encoded by]–Protein	UniProt^53^	3417	https://www.uniprot.org/
Protein–[interacts with]–Protein	STRING^14^	127767	https://string-db.org/

*ADR* adverse drug reaction, *DP* disease phenotype.

### Analysis pipeline

To determine whether ADRs and DPs representing the same phenotype share common mechanisms, we first defined the phenotypic and mechanistic similarities between ADRs and DPs. These definitions are detailed as follows.

#### Phenotypic similarity

To define phenotypic similarities, we utilized terminologies provided by the *medical dictionary for regulatory activities* (MedDRA)^15^, which provides the most comprehensive and widely used phenotypic hierarchies. MedDRA organizes terms across three levels: system organ class (SOC), preferred term (PT), and lowest level term (LLT). The SOC level represents the highest level of the MedDRA hierarchy, grouping medical terms based on etiology, anatomical site or purpose (e.g., cardiac disorders or nervous system disorders). The PT level represents distinct medical concepts (e.g., Myocardial infarction), while the LLT corresponds to the most specific level capturing individual symptoms, signs or diagnosis. LLT provide the most granular representation of clinical terminology in MedDRA.

To assess phenotypic similarity between ADRs and DPs, we considered two methods: (i) ADR–DP pairs were defined as SOC-similar if both mapped to the same SOC, indicating shared physiological or anatomical relevance; (ii) pairs were defined as LLT-similar if both shared the same LLT identifier, capturing identical clinical manifestations. We note that ADR and DP nodes in our KG were represented at the LLT level.

#### Mechanistic similarity

While ADRs and DPs are not directly connected in our KG, they are indirectly linked through their shared associated proteins. Consequently, their proximity within the graph, driven by these shared proteins, reflects their potential mechanistic relationship. Therefore, we consider the proximity of ADRs and DPs within the KG as a measure of their mechanistic similarities.

#### Graph representation and embedding evaluation via link prediction

To assess network-based proximities between nodes in our KG, we embedded the graph into a vector space using three graph representation learning methods: *node2vec*^16^, *GraphConv*^17^, and *relational graph convolutional networks* (RGCN)^18^ (Methods). These methods were chosen for their strengths in capturing structural and relational patterns in the KG: node2vec preserves both local and global connectivity using biased *random walks*; GraphConv encodes local neighborhood information via *graph convolution*s; and RGCN extends this by incorporating edge types for modeling multi-relational graphs.

To compare the embeddings generated by these models, we used a link prediction task, i.e., predicting the likelihood of an edge between two nodes, as a benchmark. Specifically, we evaluated their ability to predict four edge types: drug–ADR, drug–protein, disease–protein, and disease–DP. Using cross-validation, we split each edge type into 90% training and 10% testing. Next, the link prediction performance of the these models was evaluated using three metrics: *mean reciprocal rank* (MRR), *hit rate*, and *precision*.

As shown in Table S1 and Fig. S3, node2vec and RGCN achieved comparable performance across all edge types, consistently outperforming GraphConv. The superior performance of node2vec and RGCN highlights their effectiveness in capturing the complex relationships of the KG. GraphConv likely underperformed due to its isotropic message passing and inability to model heterogeneous and multi-relational structures. In contrast, RGCN directly incorporates edge-type information and node2vec captures local and global structure via random walks, enabling them to better represent the complexity of the KG.

#### Embedding-based biological evaluation

To further evaluate the biological relevance of the embeddings generated by node2vec and RGCN, we tested their ability to capture relationships between proteins and phenotypes.

Specifically, we utilized benchmark phenotype–protein pairs derived from prior knowledge and statistical analysis using three ADR-related databases: (i) T-ARDIS^19^, linking proteins to ADRs via drug–target relationships and statistical testing; (ii) literature-based associations for ADR–proteins^20^, curated manually from published studies; and (iii) Smit et al.^21^, combining drug pharmacokinetics with bioactivity data to infer protein–ADR associations, as well as two DP-related databases: (i) curated associations^22^, linking DPs to proteins via known gene–disease mappings, and (ii) literature-based DP–proteins associations^22^, mined using NLP techniques from PubMed abstracts (Methods). We assessed the closeness of these benchmark protein-phenotype pairs in the embedding spaces generated by each model.

A key challenge in this analysis is the non-convex nature of the model’s cost functions, which can produce slightly different embeddings due to random initialization^23,24^. To ensure robustness, we developed a pipeline to evaluate distances between ADR–proteins and DP–proteins in the embedding space robustly (Fig. 1B). To evaluate biological coherence, we assessed whether benchmark proteins were enriched among the top-ranked neighbors of their associated phenotypes using a *gene set enrichment analysis* (GSEA) framework (Fig. 1C). We then calculated the percentage of phenotypes with significantly enriched benchmark proteins in their neighborhood (adjusted *p* < 0.05). As shown in Fig. 1D, node2vec outperformed RGCN, identifying a higher percentage of significant protein–phenotype pairs, demonstrating its superior ability to preserve biologically meaningful relationships in the embedding space. Based on both link prediction and biological evaluations, we selected node2vec as the preferred method for our analysis.

### Phenotype similarity analysis

Having a measure of mechanistic similarity between ADRs and DPs, we tested the hypothesis that similar phenotypes may share similar underlying mechanisms. To evaluate this, we examined whether such pairs are significantly closer to each other in the embedding space than phenotypically dissimilar pairs (null distribution).

**Fig. 2:**
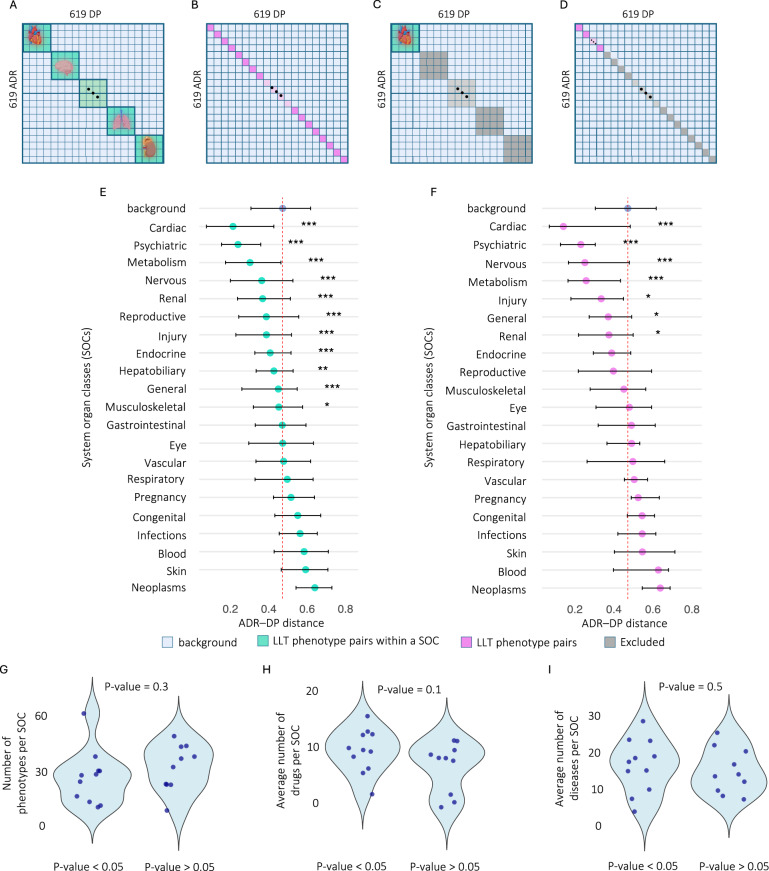
Embedding similarity analysis of phenotypes. Schematic illustration of embedding distance matrices comparing **A** all pairs of SOC-similar ADR–DPs (green) with all pairs of SOC-dissimilar ADR–DPs (blue); **B** all pairs of LLT-similar ADR–DPs (magenta) with all pairs of LLT-dissimilar ADR–DPs (blue); **C** all pairs of SOC-similar ADR–DPs related a specific SOC group (green) with all pairs of SOC-dissimilar ADR–DPs (blue); and **D** all pairs of LLT-similar ADR–DPs related a specific SOC group (magenta) with all pairs of LLT-dissimilar ADR–DPs (blue). **E**, **F** show statistical comparisons of embedding distances for SOC-similar and LLT-similar ADR–DPs, respectively, across different SOC groups. Null distributions for SOC-dissimilar and LLT-dissimilar ADR–DPs are shown in blue. Embedding distances are represented by the rank of cosine similarities across 1000 runs, with lower rank values indicating higher similarity. Violin plots comparing **G** the number of phenotypes, **H** the average number of related drugs, and **I** the average number of related diseases per SOC between SOCs with *p* < 0.05 and the remaining SOCs (Wilcoxon rank-sum test, *α* = 0.05).

We considered two definitions of similarity: SOC-similarity (Fig. 2A) and LLT-similarity (Fig. 2B). The corresponding null distributions were constructed using SOC-dissimilar and LLT-dissimilar ADR–DP pairs, respectively. We then used a one-sided *Wilcoxon* test to assess the significance of embedding similarities between all pairs of phenotypically similar ADR–DPs and null distributions. Notably, we excluded SOCs with less than five phenotypes represented in our KG, resulting in 21 SOCs and 619 LLTs ADRs and DPs.

In our statistical analysis, we obtained p-values of $6\times {10}^{-21}$ and $3\times {10}^{-3}$ for SOC and LLT similarities, respectively, demonstrating that phenotypically similar pairs are significantly closer in the embedding space compared to dissimilar pairs. Nonetheless, these findings raise the question of whether this observed significance applies uniformly across all phenotypes or is disproportionately influenced by specific phenotype groups. This issue will be explored in the subsequent section. LLT phenotype pairs

### System organ class (SOC) similarity-based analysis

We examined whether our hypothesis holds within specific organ systems. To this end, we grouped ADR–DP pairs by their SOC and investigated whether ADRs and DPs associated with the same SOC share similar mechanisms. Specifically, we calculated embedding similarities for all ADR–DP pairs within each SOC and compared to a null distribution of SOC-dissimilar phenotype pairs (Fig. 2C). Results for all 21 SOCs are shown in Fig. 2E and Table 3. Of the 21 SOCs analyzed, 11 showed significant embedding similarities, suggesting that the observed mechanistic overlap is organ-specific.

**Table 3:** *P* values representing the significance of embedding similarities for SOC-similar ADR–DPs across different system organ class (SOC) groups

SOC	Statistics	Statistics for each SOC using the original KG	Statistics for each SOC using the reduced KG	Statistics for each SOC after confounder removal using the reduced KG
Nervous	*P* value adjusted *p* value effect size	$$\begin{array}{ll}4.7\times {10}^{-161}\\ 9.9\times {10}^{-160}\\ -0.44\end{array}$$	$$\begin{array}{l}2.7\times {10}^{-95}\\ 1.4\times {10}^{-94}\\ -0.36\end{array}$$	$$\begin{array}{l}1.0\\ 1.0\\ 0.29\end{array}$$
Psychiatric	*P* value adjusted *p* value effect size	$$\begin{array}{l}2.9\times {10}^{-148}\\ 3.08\times {10}^{-147}\\ -0.94\end{array}$$	$$\begin{array}{l}1.3\times {10}^{-144}\,\\ 1.4\times {10}^{-143}\,\\ -1.0\end{array}$$	$$\begin{array}{l}1.2\times {10}^{-08}\\ 4.2\times {10}^{-08}\,\\ -0.36\end{array}$$
Metabolism	*P* value adjusted *p* value effect size	$$\begin{array}{c}1.4\times {10}^{-136}\\ 1.0\times {10}^{-135}\\ -0.67\end{array}$$	$$\begin{array}{l}1.7\times {10}^{-151}\\ 3.7\times {10}^{-150}\\ -0.72\end{array}$$	$$\begin{array}{l}2.6\times {10}^{-102}\\ 1.7\times {10}^{-101}\,\\ -0.66\end{array}$$
Cardiac	*P* value adjusted *p* value effect size	$$\begin{array}{l}4.0\times {10}^{-134}\\ 2.1\times {10}^{-133}\\ -0.92\end{array}$$	$$\begin{array}{l}1.0\times {10}^{-131}\\ 7.3\times {10}^{-131}\\ -0.9\end{array}$$	$$\begin{array}{l}5.1\times {10}^{-58}\\ 2.7\times {10}^{-57}\\ -0.66\end{array}$$
Renal	*P* value adjusted *p* value effect size	$$\begin{array}{l}6.7\times {10}^{-26}\\ 2.8\times {10}^{-25}\\ -0.37\end{array}$$	$$\begin{array}{l}1.0\times {10}^{-21}\\ 4.3\times {10}^{-21}\\ -0.37\end{array}$$	$$\begin{array}{l}1.6\times {10}^{-09}\\ 5.5\times {10}^{-09}\\ -0.26\end{array}$$
Reproductive	*P* value adjusted *p* value effect size	$$\begin{array}{l}1.0\times {10}^{-11}\\ 3.6\times {10}^{-11}\\ -0.28\end{array}$$	$$\begin{array}{l}7.1\times {10}^{-08}\\ 2.1\times {10}^{-7}\\ -0.22\end{array}$$	$$\begin{array}{l}2.5\times {10}^{-05}\\ 8.3\times {10}^{-05}\\ -0.23\end{array}$$
Endocrine	*P* value adjusted *p* value effect size	$$\begin{array}{l}9.9\times {10}^{-04}\\ 2.3\times {10}^{-3}\\ -0.21\end{array}$$	$$\begin{array}{l}7.2\times {10}^{-04}\\ 1.5\times {10}^{-03}\\ -0.24\end{array}$$	$$\begin{array}{l}8.0\times {10}^{-02}\\ 0.16\\ -0.09\end{array}$$
Hepatobiliary	*P* value adjusted *p* value effect size	$$\begin{array}{l}8.4\times {10}^{-03}\\ 1.7\times {10}^{-02}\\ -0.2\end{array}$$	$$\begin{array}{l}3.0\times {10}^{-05}\\ 7.1\times {10}^{-05}\\ -0.37\end{array}$$	$$\begin{array}{l}1.5\times {10}^{-05}\\ 3.6\times {10}^{-05}\\ -0.38\end{array}$$
Musculoskeletal	*P* value adjusted *p* value effect size	$$\begin{array}{l}2.0\times {10}^{-02}\\ 4.7\times {10}^{-02}\\ -0.05\end{array}$$	$$\begin{array}{l}0.4\\ 0.7\\ -0.002\end{array}$$	$$\begin{array}{l}0.9\\ 1.0\\ 0.07\end{array}$$

Results are shown for the original knowledge graph (KG), the reduced KG, and after excluding confounding drugs from reduced KG.

To assess whether this significance could be biased by node density, we compared the number of phenotypes per SOC as well as the average number of their related drugs and diseases, between the 11 significant SOCs and the remaining 10 (Fig. 2G–I). No significance differences were found (Wilcoxon rank-sum test, *α* = 0.05), suggesting that the observed results reflect biological signal rather than differences in graph density.

### Low-level term (LLT) similarity-based analysis

We further evaluated LLT similarities of ADR–DP pairs and grouped phenotypes by their respective SOC to determine whether LLT-similar ADR–DPs within the same SOC share common mechanisms. To test this hypothesis, we compared the similarity distribution of LLT-similar ADR–DPs within each SOC to a null distribution of LLT-dissimilar phenotype pairs (Fig. 2D). The results presented in Fig. 2F, indicate that phenotypes associated with specific organ systems, such as cardiac, psychiatric, nervous system, and metabolism categories, exhibit significantly higher similarities compared to the null distribution. In addition, injury, general and renal disorders showed borderline significance (one-sided Wilcoxon test *p* value less than 0.05).

**Fig. 3:**
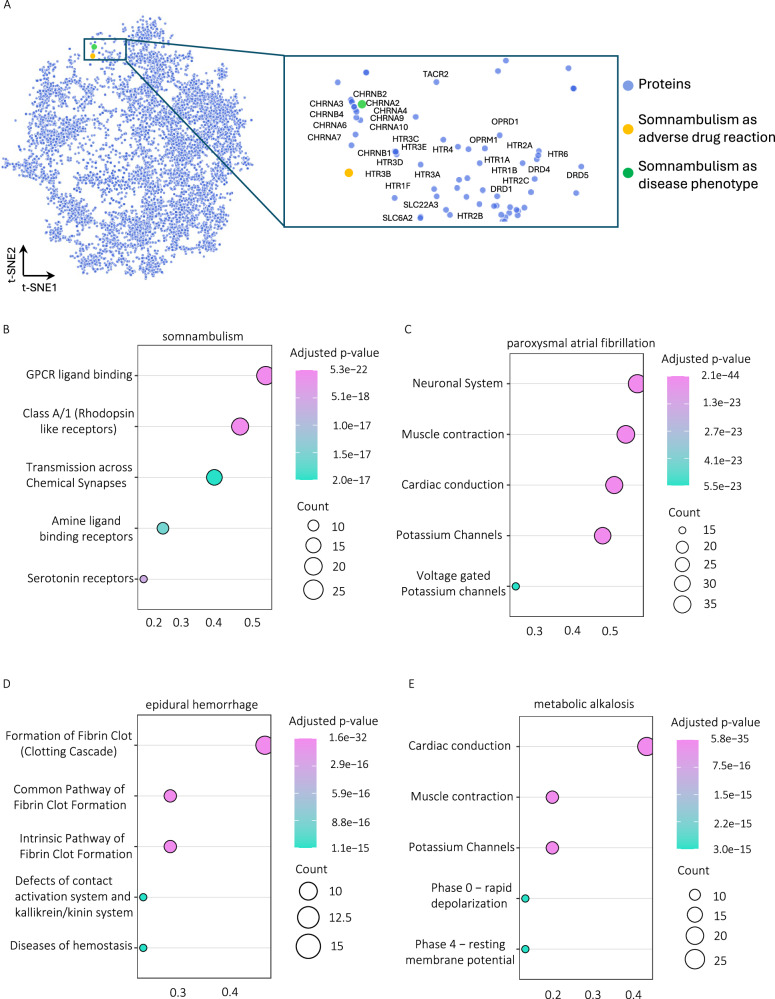
Enriched pathways for proteins with highest similarities to LLT-similar adverse drug reaction (ADR) and disease phenotype (DP) pairs. **A** t-SNE of the embedding space showing the proximity of somnambulism ADR (yellow) and DP (green). The proteins (blue) clustered around these points, highlights their association with somnambulism. Enriched pathways for **B** Somnambulism (psychiatric disorders), **C** Paroxysmal atrial fibrillation (cardiac disorders), **D** Epidural hemorrhage (Injury), and **E** Metabolic alkalosis (metabolism disorders).

The high embedding similarities observed between SOC- and LLT-similar phenotypes are induced by their shared related proteins in the network. Therefore, proteins that are close to these paired phenotypes in the embedding space are expected to represent the common mechanism of ADRs and DPs. To investigate this hypothesis, we focused on the top phenotypes within SOCs that showed significant *p* values in our LLT similarity-based analysis and explored their related proteins. To identify such proteins close to both ADR and DP, we applied a GSEA-inspired approach (Methods). For psychiatric disorders, a t-SNE plot illustrates the proximity of somnambulism (sleepwalking) ADR and DP in the embedding space, with associated proteins clustering nearby (Fig. 3A). We further determined the biological functions of identified proteins through an over-representation analysis using Reactome pathways^25^. The enriched pathways, representing the underlying mechanisms of these top phenotype pairs, are represented in Fig. 3B–E and Table S2. For psychiatric disorders, these proteins form the basis of the analysis of enriched somnambulism-based pathways,including class A/1 (Rhodopsin-like) receptors, such as melatonin receptors, which regulate circadian rhythms and sleep-wake cycles (Fig. 3B)^26–28^. These receptors influence the body’s internal clock by modulating melatonin production, which increases during sleep to promote rest. Disruptions in this pathway can disturb the normal suppression of motor activity during sleep, resulting in episodes of sleepwalking. Serotonin signaling was also enriched, supporting the serotonergic hypothesis of sleepwalking^29^. Serotonin interacts with neurons during sleep to maintain postural control. When the system is dysregulated, it may fail to inhibit motor activities during sleep, which also explains the motor behavior characteristic of sleepwalking^30^.

In the case of cardiac disorders, paroxysmal atrial fibrillation is associated with pathways involving cardiac conduction, potassium channels, neuronal system, muscle contraction, and voltage gated potassium channels, reflecting its core electrophysiological mechanisms (Fig. 3C). For example, altered potassium channel activity shortens the action potential duration, disrupting depolarization and promoting reentry circuits. This impairs electrical signal propagation, and coordinated cardiac conduction and muscle contraction^31,32^. For epidural hemorrhage (Injury), the most enriched pathways include disease of hemostasis, fibrin clot formation, and the clotting cascade, reflecting vascular injury and coagulation dysfunction characteristic of hemorrhagic events (Fig. 3D)^33,34^. Additionally, defects in the contact activation system and kallikrein/kinin system contribute to exacerbating bleeding and inflammation in hemorrhagic events^35^.

For metabolic alkalosis (metabolism disorders), the enriched pathways include cardiac conduction, muscle contraction, potassium channels, rapid depolarization (phase 0), and resting membrane potential (phase 4) (Fig. 3E), reflecting the downstream neuromuscular effects of electrolyte imbalances caused by alkalosis, in particular hypokalemia^36^. Hypokalemia disrupts potassium ion gradients, impairing channel activity and delaying cardiac repolarization, often leading to symptoms like muscle weakness and arrhythmias^37^. For instance, low extracellular potassium ions shift the cell’s membrane potential to a more negative state, requiring stronger stimuli for depolarization. This cascade disrupts normal heart rhythms and weakens muscle function.

The pathways enriched for each phenotype within the significant SOCs align with relevant biological mechanisms, further supporting that the molecular pathways shared between ADRs, and DPs are not just statistically significant but also biologically coherent.

### Sensitivity analysis

To assess the robustness of our findings, we examined whether the significance observed for the 11 SOCs in the previous section persists under variations in the underlying data or representation strategies. Specifically, we conducted a sensitivity analysis to identify which SOCs remain consistently significant across three alternative scenarios: (i) variation in source dataset, (ii) variation in the dataset versions, and (iii) variation in levels of phenotype representation within the MedDRA hierarchy. This analysis aims to determine which SOC-specific findings are stable and not driven by dataset-specific artifacts or modeling choices.

#### Variation in source dataset

**Fig. 4:**
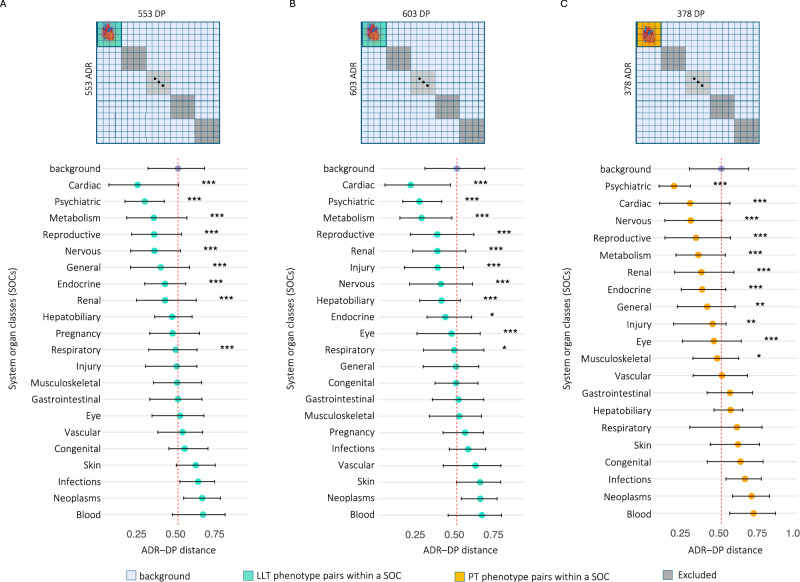
Sensitivity analysis of SOC-level ADR–DP similarities under three different knowledge graph (KG) constructions. Bar plots show the distribution of embedding distances between ADR–DP pairs grouped by system organ class (SOC) under three conditions: **A** using disease–gene associations from the Comparative Toxicogenomics Database instead of DisGeNET; **B** using an older version of drug–protein data (DrugBank 2022 v) instead of the current DrugBank–DrugCentral combination; **C** Representing phenotypes at the Preferred Term level instead of the Lowest Level Term level. In each panel, the top matrix shows the distance between ADR and DP embeddings for the included phenotypes. The bottom dot plots compare the average ADR–DP embedding distances within each SOC (colored dots) to the background distribution (gray bars) across 21 SOCs. Significance levels are denoted as ***p < 0.001, **p < 0.01, and *p < 0.05 (Wilcoxon rank-sum test, one-sided).

To test the generalizability of our findings across different sources of disease–gene associations, we reconstructed the KG by replacing DisGeNET data with curated associations from the Comparative Toxicogenomics Database (CTD)^38^. CTD is a manually curated resource that integrates molecular and toxicological data of chemicals, genes, phenotypes, diseases, and environmental exposures. After replacing DisGeNET^13^ with CTD^38^, we observed that only 38% of disease–gene links overlapped between the two sources, and 56% of disease nodes were removed from the KG. Despite these substantial changes, the set of significantly enriched SOCs remained largely consistent. Specifically, 8 out of the 11 SOCs retained their significance (Fig. 4A), suggesting the robustness and biological relevance of our findings across distinct data sources.

#### Variation in the dataset versions

Given the evolving nature of KGs, conclusions drawn from them may inherently depend on the completeness of the data at the time of construction. To investigate the sensitivity of our findings to such changes, we evaluated the impact of using a different version of drug–protein interaction data on the stability of SOC-level significance. Specifically, we replaced the current DrugBank–DrugCentral combination with an earlier version of DrugBank (2022 v), resulting in a 42% reduction in drug–protein links within the KG. Despite these substantial reductions, the set of significantly enriched SOCs remained largely stable. Notably, nine out of the original 11 SOCs retained their statistical significance (Fig. 4B), suggesting that our results, to a high degree, are robust to the specific version of data employed.

#### Variation in levels of phenotype representation

To investigate the sensitivity of the results for each SOC to the granularity of clinical terminology, we constructed an alternative KG using PTs instead of LLTs to represent ADRs and DPs. This replacement resulted in a decrease of phenotype nodes from 619 to 378, reflecting a 39% reduction of the clinical terminology used. Despite this reduction, the similarity analysis performed on the PT-level yielded results largely consistent with those from the LLT representation. Specifically, 10 out of the original 11 significantly enriched SOCs retained their significance (Fig. 4C). This demonstrates that our findings are robust across different levels of the MedDRA hierarchy.

### Robustness against confounding effects

According to the principles of drug discovery and repurposing, drugs are often developed or repurposed to target proteins or pathways implicated in the genetic basis of diseases. As a result, the proximity of ADRs and DPs in the embedding space and statistical significance observed in our analysis may be influenced by the genetic associations of DPs, which inherently reflect their GWAS-based origins, and their alignment with the therapeutic indications of the drugs. Hence, it is essential to ensure that the observed statistical significance arising from mechanistic overlaps between ADRs and DPs are robust against the biases introduced by drug indication effects.

A potential method to investigate the impact of this confounding effect for each SOC is to exclude drugs that have both ADRs and therapeutic indications within the same SOC from our analysis. To identify therapeutic indication SOCs for drugs, we used the data provided by a previous study^39^. Among 409 drugs in our KG, the therapeutic indication SOCs for 302 drugs were available in their dataset, which resulted in a reduction of 26% of drugs from our KG (denoted as the reduced KG). Notably, their database covered all the SOCs for which we observed significant results (Fig. 2E, F), except for the general category, which we then excluded from our analysis. Additionally, we excluded the Injury SOC, as it was left with only four phenotypes in the reduced KG, which was below the threshold of five phenotypes used in the *phenotype similarity analysis* section.

We assessed the robustness of our results against the 26% reduction in drugs prior to investigating confounding effects. To this end, we repeated the network representation learning on the reduced KG to find the embeddings and performed statistical analysis. The results of statistical significance for each SOC are summarized in Table 3. Interestingly, the statistical significance for all SOCs, except for the musculoskeletal domain, which already had marginal significance, remained unchanged. These results suggest that our analysis is robust even after the removal of a substantial number of links from the network.

Next, for each SOC, we excluded drugs that had both therapeutic indications and ADRs within the same SOC (referred to as potential confounding drugs) and repeated our analysis pipeline to find the embeddings. Table S3 presents the number of phenotypes after excluding potential confounding drugs per SOC. Statistical significance before and after removing these drugs is shown in Table 3. Despite the removal of potential confounding drugs from the KG, six of eight SOCs retained significant *p* values, indicating that the observed similarities of ADR–DP pairs within the same SOC are robust and primarily driven by shared mechanisms rather than solely by the genetic associations underlying the therapeutic indications. The endocrine and nervous systems were the two SOCs that lost significance, which can be attributed to two factors^1^: the confounding influence of therapeutic indications, where the genetic associations underlying DPs overlap with the therapeutic targets of drugs; and^2^ the substantial reduction in KG size after removing confounding drugs, which decreases the number of links available for capturing mechanistic relationships.

To assess the latter, we evaluated the extent to which the exclusion of potential confounding drugs for each SOC impacted the statistical significance of other SOCs. As summarized in Table S4, the removal of confounding drugs for all SOCs (including endocrine), except for the nervous system, did not significantly affect the statistical significance of other SOCs, suggesting that the results are largely robust to the removal of confounding drugs. In contrast, excluding these drugs for the nervous system led to significant changes in the statistical significance of other SOCs. Further analysis of the percentages of excluded drugs and drug–ADR links for each SOC (Table S5) revealed that the nervous system had the highest percentage of removed drugs (28%) and drug–ADR links (59%) among all SOCs. This finding indicates that the loss of significance for the nervous system is likely due to the substantial reduction in its connectivity within the KG, disrupting its mechanistic relationships. Additionally, drugs used to treat nervous system disorders are highly interconnected with ADRs across multiple SOCs, impacting the overall structure of the KG and influencing results of other SOCs. The biological basis for this observation may stem from the fact that the nervous system is highly complex and tightly regulated. Therefore, drugs indicated for nervous system disorders target specific neural pathways, such as neurotransmission and synaptic or hormonal signaling, which are shared across multiple organ systems. In addition, they can inadvertently affect other pathways due to the interconnected nature of neural networks. Consequently, there is a high overlap between drugs intended for neurological indications and those causing neurological ADRs. Removing these confounding drugs significantly reduces the links in the KG for this SOC, impacting the statistical significance of our results.

For endocrine, although only a small proportion of drugs (3%) and drug–ADR links (10%) were removed from the KG, the associated *p* value lost its significance. Moreover, this removal did not affect the significance of p-values for other SOCs. These observations increase the likelihood that the observed significance in the endocrine system is driven more by drug indications than by ADR-related mechanisms.

We further investigated whether the observed ADR–DP similarities within certain SOCs are driven by the specific class of drugs used in those categories. To address this, we assessed the enrichment of drugs within known pharmacological classes for the six SOCs that retained statistical significance after removing potential confounding drugs. To this end, we classified the drugs based on their mechanisms of action using second-level *anatomical therapeutic chemical* (ATC) classification codes^40^. Then, we applied a *Fisher’s exact* test to compare the proportion of drugs linked to a specific SOC and a specific ATC class to the proportion of remaining drugs in the same ATC category. Interestingly, none of the drug classes were significantly enriched in any of the SOCs (BH adjusted *p* value < 0.05), except for *N06-Psychoanaleptics* class enriched in *psychiatric* disorders (Table S6). This finding suggests that the observed ADR–DP similarities are unlikely to be driven by drug class biases. Instead, these associations are more likely to reflect underlying biological mechanisms shared between ADRs and DPs related to each SOC.

## Discussion

In this study, we investigated the hypothesis that phenotypically similar ADRs and DPs share underlying molecular mechanisms. Our findings support this hypothesis, particularly within certain organ systems. To evaluate the robustness of these findings, we conducted multiple sensitivity analyses across varying data sources, dataset versions, and phenotype representations. Notably, the majority of significantly enriched SOCs remained consistent across these variations, indicating that our results are, to a high degree, robust against dataset-specific artifacts or modeling choices. In addition, we showed that therapeutic indications of drugs do not have a significant impact on the observed associations.

While existing efforts provide valuable insights into ADR mechanisms^41^, the mechanisms behind many ADRs remain unknown. By integrating a dual perspective of drug-induced and disease-related phenotypes, this study introduces a novel framework for mechanistically linking ADRs and DPs, addressing a critical gap in understanding the molecular drivers underlying clinical manifestations. Such insights are crucial, not only for improving patient safety and healthcare outcomes, but also for advancing our understanding of disease biology and therapeutic mechanisms. Early identification of these mechanisms could prevent the approval or release of drugs likely to cause specific ADRs, thereby reducing the risk of adverse events in clinical practice.

Our findings suggest that drugs targeting proteins in proximity to those associated with DPs in specific SOCs may be more likely to cause ADRs with similar phenotypes. For example, targeting proteins related to DPs in the cardiac, psychiatric, metabolism, renal, reproductive and hepatobiliary categories may increase the likelihood of inducing ADRs with overlapping manifestations. Furthermore, identifying mechanistic similarities between phenotypes can enhance our understanding of why certain drugs cause similar ADRs, potentially aiding safety assessments in early development stages. We present this framework as a hypothesis-generating tool that offers a promising direction for anticipating ADR risks. However, translating mechanistic similarity into actionable safety decisions will require further validation, such as retrospective drug safety analyses or incorporation into prospective screening pipelines.

DP associated proteins are solely derived from GWAS-related data, which presents a limitation as it captures genetic variations without accounting for gene-environment interactions or activation mechanisms. For certain organ systems, such as gastrointestinal, infections, skin, immune-related, and respiratory disorders, we did not observe significant ADR–DP mechanistic similarities. This lack of significance can be attributed to the inherent complexity and multifactorial nature of these organ systems. They encompass a wide range of phenotypes influenced by genetic, environmental, and immune factors, leading to diverse mechanistic profiles that obscure shared mechanisms between ADRs and DPs. For example, gastrointestinal and respiratory disorders are heavily influenced by gene-environment interactions, including factors such as diet, microbiome composition, allergens, and pollutants. Similarly, phenotypes associated with skin, gastrointestinal and respiratory systems primarily function as barrier tissues that react to external stimuli like pathogens, allergens, and environmental toxins. This reactivity complicates their mechanistic alignment with DP data, which often focuses on intrinsic genetic drivers. Furthermore, neoplasm, immune, and infection categories present additional challenges in identifying shared ADR–DP mechanisms due to their inherent heterogeneity. Many neoplasms are not exclusively genetically driven but arise from complex combinations of genetic, environmental, and stochastic factors, making it difficult to pinpoint shared mechanisms with DPs. Similarly, infections involve external pathogens that elicit host responses depending on the pathogen type and tissue environment, which are not always linked to intrinsic molecular mechanisms. These observations suggest that incorporating additional layers of information, such as gene expression or proteomics profiles may enhance the explanatory power of the KG, enabling the detection of mechanistic associations even for more complex SOCs.

Organ systems such as cardiac, psychiatric, nervous systems rely on well-defined, tightly regulated molecular pathways that are particularly vulnerable to both genetic disruptions and pharmacological interactions. For example, in cardiac SOC, ion channels and transporters such as potassium and sodium channels, critical in arrhythmias, are both drug targets and implicated in genetic cardiac disorders. In psychiatric SOC where neurotransmitter receptors like serotonin and dopamine receptors are common drug targets and have genetic associations with psychiatric conditions, or neurological SOC which often involve glutamate or GABA pathways, play key roles in genetic conditions like epilepsy and are also targeted by pharmacology, making them prone to overlap in both protein-based ADRs and gene-based DPs.

While our study provides valuable insights, certain limitations should be acknowledged to guide future research. One key challenge is the inherent incompleteness of the KG, i.e., the existence of missing links, which may influence the accuracy of mechanistic predictions. For example, some ADRs or DPs may be extremely underrepresented in terms of their connections to drugs or diseases, potentially affecting their relative position and similarity in the embedding space. This is particularly evident in the congenital SOC, where each ADR is, on average, connected to only 1.09 drugs (Fig. S4). This extreme underrepresentation could also be attributed to the rarity of congenital phenotypes, which are less frequently reported or associated with drugs, further limiting their representation in the KG. Additionally, some ADRs are mediated by reactive metabolites that have distinct structures and activities compared to the parent drug^42^. For example, Lee et al.^43^, demonstrated that incorporating metabolite structures significantly improves ADR predictions, particularly for renal disorders. However, our KG does not include information on drug metabolite-protein interactions due to the lack of comprehensive datasets. Furthermore, while we used MedDRA as the primary ontology for clinical phenotypes, given its wide adoption in ADR research and inclusion in key resources like SIDER, alternative clinical ontologies such as SNOMED-CT may offer a compelementary resource for ADRs and DPs. Future efforts to integrate SNOMED-CT could enhance the clinical relevance of the KG. Additionally, while our analysis focuses on phenotypes grouped by SOCs, it does not account for the biological and clinical heterogeneity within individual SOCs. Addressing this intra-SOC variability could further enhance the granularity and generalizability of future findings. Finally, while our study leverages SOCs to group phenotypes, it is important to acknowledge the inherent limitations of using SOCs as they are human-defined clinical classifications that may not always align with underlying molecular mechanisms. Similar phenotypes classified under the same SOC can arise from entirely different molecular events, highlighting the concept of divergent phenotypes, where distinct genomic contexts and pathways lead to similar clinical outcomes. For example, long QT syndrome, where a prolonged QT interval on an electrocardiogram, can arise from mutations in *KCNQ1*^44^, impairing potassium efflux, or *SCNA5*^45^, causing persistent sodium influx, both delaying cardiac repolarization. Conversely, shared molecular mechanisms can drive convergent phenotypes, where a single molecular pathway results in varying clinical presentations across different SOCs. For example, mutations in *PTEN* gene, a key regulator of the PI3K-AKT signaling pathway, are linked to diverse phenotypes, including cancer^46^, obesity^47^ and Alzheimer’s disease^48^. This discrepancy highlights the challenges of relying solely on symptom-based classification systems, which may oversimplify the complex and multifaceted biological processes involved in ADRs and DPs. Therefore, while SOCs provide a useful framework for organizing clinical data, integrating molecular and mechanistic insights is crucial for a more comprehensive understanding of the relationships between ADRs and DPs.

Our study highlights the potential of integrating ADR and DP data to uncover shared molecular mechanisms. By identifying connections between ADRs and DPs, we show that phenotypic similarity can inform mechanistic insights. Specifically, we demonstrated that for certain SOCs, phenotypically similar ADRs and DPs exhibit significantly closer mechanistic relationships. These findings open new avenues for future network-based investigations into phenotype-driven drug safety and mechanism discovery. To support further research and reproducibility, our analysis pipeline and KG are publicly available on GitHub (https://github.com/faren-f/ADR_DP_similarity).

## Methods

### Knowledge graph construction

We constructed a comprehensive KG by integrating multiple node types, including drugs, diseases, proteins, ADRs, and DPs. Table 1 summarizes nodes, their counts in our KG, and their data sources. Links between these node types were derived from various sources, as outlined in Table 2, which provides an overview of the edge types and their count and their corresponding sources. Except for the STRING PPI network, all the edges in our KG were retrieved from *NeDRexDB* (version:2)^49^, an integrative and up-to-date platform designed for drug repurposing and disease module discovery. A detailed description of all data sources used in this study is provided below.

*Drug–ADR associations* were collected from the latest version of the *SIDER* (SIDER 4, version: 4)^10^, a widely used public database containing 140,064 drug–ADR relationships extracted from drug labels and enhanced through text mining tools. To ensure consistency across datasets, ADRs were mapped to standardized MedDRA terms using the LLT identifiers. For drugs, we used DrugBank identifiers. We retrieved this link from *NeDRexDB* on 02 April 2023. To improve data quality, we excluded drug–ADR links where the ADR matched the drug’s indication in our dataset, as these may reflect rare or unusual cases, or potential false positives.

*Disease–DP associations* were obtained from the *human phenotype ontology* (HPO) database (version: v2023-04-05)^11^, which provides an extensive catalog of phenotypic abnormalities linked to human diseases. We retrieved this link from *NeDRexDB* on 02 April 2023. Each DP is represented by a unique HPO identifier, and each disease is identified using a MONDO identifier.

Notably, we excluded ADRs and DPs linked to a large number of drugs and diseases, such as nausea, headache, and seizure to prevent bias introduced by non-specific or frequent associations. To define “very frequent” ADRs and DPs, we applied a data-driven thresholding approach based on ~10% of the maximum observed frequencies. For ADRs, the maximum frequency was 453, suggesting a threshold of 45; for DPs, the maximum was 917, corresponding to a threshold of 92. We conservatively rounded these values up to 50 and 100, respectively, to retain more information (Fig. S1).

*ADR–DP mapping* was performed using phenotype cross-references provided by BioPortal (version: v2023-04-02)^8^, which integrates biomedical ontologies and their semantic alignments. We retrieved all cross-referenced mappings between MedDRA and HPO terms from *NeDRexDB* on 02 April 2023. Form the initial set of 1240 ADR–DP mappings, we retained only those where the ADR was linked to at least one drug (via SIDER) and the DP was associated with at least one disease (via HPO). This filtering step resulted in 649 ADR–DP pairs used in the KG.

*Drug–target associations* were incorporated into our KG using data from two resources: *DrugBank* (version: 5.1.11)^12^, a comprehensive database of pharmaceutical knowledge, and DrugCentral^50^, a curated database of drug mechanisms and targets. These associations are integrated via NeDRexDB. We retrieved the combined drug–target links from *NeDRexDB* on 23 October 2023, where drugs are represented using DrugBank identifiers and proteins with Uniprot identifiers. We included only those drugs that had at least one target listed in DrugBank and at least one ADR recorded in SIDER. Notably, access to DrugBank’s dataset is restricted and requires a premium license^12^.

*Disease–gene associations* were obtained from *DisGeNET* database^13^, an expert-curated resource linking human diseases to their associated genes. These associations were retrieved via *NeDRexDB* on 02 April 2023, where diseases are represented using MONDO identifiers and genes with Entrez identifiers. We retained only diseases with at least one gene association in DisGeNET and at least one phenotype-related association in HPO. Mapping from genes (Entrez identifier) to proteins (UniProt identifier) is accessible via NeDRexDB.

*Protein–protein interactions* were collected from the *STRING* database (version: 12.0)^14^, a comprehensive resource for experimental and predicted protein–protein interaction data. To ensure high-confidence interactions, we applied a confidence score threshold of 800 to filter out lower-confidence links. The data were retrieved from https://string-db.org/ on 22 December 2023.

An overview of the step-by-step framework used to construct and preprocess our KG is provided in Fig. S2, adapted from our previous study^6^.

### Benchmark datasets

We used benchmark datasets that provide information on proteins and associated ADRs (or DP) obtained from previous studies to evaluate and compare our embedding spaces that are derived from different models. Specifically, we used three ADR-related databases:

- *T-ARDIS*^19^. This dataset identifies statistically significant associations between proteins and ADRs by combining information from drug–protein and drug–ADR databases. The data are available in http://www.bioinsilico.org/T-ARDIS. We identified 252 ADRs common to our KG and the T-ARDIS dataset.
- *Literature-based associations for ADR–proteins*. Kuhn et al.^20^ obtained known protein–ADR pairs, derived from a survey of the literature. We identified 41 ADRs common to our KG and the Kuhn dataset.
- Smit et al.^21^, provides statistically significant associations between protein targets and ADRs by integrating drug–protein bioactivity data with ADR reports from the FAERS and SIDER databases. These associations account for drug pharmacokinetics by using the ratio of unbound plasma drug concentration to in vitro potency to identify likely target-ADR relationships. We identified 35 ADRs common to our KG and the Smit dataset.

And two DP-related databases:

- *Curated Associations*. Several DP terms are equivalent to disease terms, allowing genes associated with these diseases to be considered as a priori known proteins for the corresponding DPs. Considering this equivalence, Lu et al.^22^ compiled a set of DP–gene associations from phenotype-genotype association databases, referred to as curated data. We identified 110 DPs that are shared between our KG and curated associations dataset.
- *Literature-based associations for DP–proteins*. Lu et al.^22^ compiled DP-related proteins by integrating data from PubMed and SemMed databases using a combination of natural language processing (NLP) methods and manual curation. Their approach employed NLP to identify co-occurrences of DP and protein keywords within abstract texts published prior to January 2022. In our analysis, we identified 129 phenotypes that overlapped between their dataset and our KG.

### Knowledge graph representation learning

We selected *node2vec* and two *graph neural network autoencoders*, namely GraphConv, and RGCN, to embed our KG since each offers distinct advantages for capturing network-based proximities and relationships.

- *Node2Vec*. Node2vec^16^ is a shallow network embedding model that learns node representations by sampling sequences of nodes through biased random walks, thereby preserving structural and neighborhood network proximity. This method is particularly suited for large-scale graphs, where preserving local and global connectivity patterns is essential. For our implementation, we utilized the Node2Vec v0.5.0 Python package. The Node2Vec hyperparameters—walk length, walks per node, hidden dimensions, and window size—were set to 100, 100, 15, and 5, respectively, to minimize the Node2Vec loss on the training set.
- *Graph neural network autoencoder*. A graph *neural network* autoencoder consists of two components: an encoder and a decoder^51^. The encoder represents nodes into a vector space, while the decoder reconstructs the adjacency relationships in the input network. For the encoder, we considered two methods^1^: *GraphConv*, a graph convolutional neural network (GCNN) that uses message passing to aggregate and encode local features from neighboring nodes, and^2^ RGCN, a GCNN designed for heterogeneous networks^18^, i.e., networks with different types of nodes and edges. For the decoder, we applied a cosine similarity layer. To implement the graph autoencoder, we used the PyTorch Geometric v2.5.3 Python package. The hyperparameters for the GraphConv model included the number of hidden channels, number of layers, and activation function for the hidden layers, which were set to 15, 3, and rectified linear unit (ReLU), respectively. For RGCN, the corresponding hyperparameters were set to 200, 3, and ReLU. All hyperparameters were optimized to minimize the training loss.

### Random KG generation

To evaluate whether the network embeddings capture biologically meaningful information, we generated embeddings for a randomly constructed KG and compared the results to those derived from the original KG. The random KG was created by shuffling the links between nodes in the original graph, specifically the drug–ADR, disease–DP, drug–protein, and disease–protein edges, while preserving the original node and edge counts.

### Robustify the embedding space

To stabilize the results of network representation learning against random initializations, we developed a pipeline to robustify the distances in the embedding space. Specifically, we trained each representation learning model 1000 times with different parameter initializations and calculated cosine similarities between ADR–proteins, DP–proteins and ADR–DPs in each run. For each ADR–DR link, we obtained their rank across all other ADR–DR links and then calculated the rank-mean across all runs. For ADR–proteins and DP–proteins links, we ranked proteins for all ADRs and DPs, respectively, and then obtained their rank-sum across all runs. The rank-mean values were used as similarity measures between the corresponding node types, where smaller ranks indicated greater similarity.

### Measures of link prediction performance

To evaluate the performance of the network representation learning models, we employed three metrics: *mean reciprocal rank* (MRR), *Precision@k*, and *Hit@k*. For all metrics, we first calculated the cosine similarity between node pairs and ranked them. MRR is defined as the mean of the reciprocal ranks of the positive links. Since lower ranks indicate higher similarity, a larger MRR value reflects better link prediction performance. Precision@k is calculated by selecting the top k links and determining the ratio of positive links to k. This metric evaluates how well the top-ranked links are enriched with positive links. Hit@k is computed by taking the top n links that include k negative links and then determining the ratio of positive links to the total number of links within this subset. These metrics provide a comprehensive assessment of the model’s ability to accurately predict links in the network.

### Protein–phenotype enrichment analysis

To evaluate the enrichment of a protein set around a certain phenotype (either ADR or DP), we performed a statistical analysis based on GSEA. Specifically, for each phenotype, we ranked proteins according to their embedding similarity to the corresponding phenotype. We then used the *fgsea* v1.28 *R* package to calculate the enrichment *p* value. This *p* value quantifies the significance of a protein set being closely associated with a given phenotype in the embedding space.

### Identifying overlapping proteins

To identify proteins associated with each phenotype pair (a phenotypically similar ADR–DP pair), we found proteins that are close to both ADR and DP. For this, we adopted an approach inspired by GSEA^47^. For each ADR, we incrementally selected its top-k closest proteins, where k ∈ {50, 100, …, 500}. We then applied GSEA to evaluate the enrichment of these top-k proteins being closely associated with the ADR’s paired DP. For each k, we calculated a *p* value and an enrichment score (ES). The optimal k was determined at the first elbow point of the ES-k curve, where the *p* value was less than 0.05. To find the elbow point, we used the *kneedle* algorithm^50^. This process was repeated for each DP phenotype to identify its respective best top-k proteins. The final step involved identifying overlapping proteins by finding the intersection of the top-k protein lists for each ADR and its paired DP. These shared proteins were subsequently analyzed using over-representation analysis of the Reactome database to identify associated biological pathways.

## Supplementary information

Supplementary Information

## Data Availability

The ADR–DP, drug–ADR, gene–disease, gene–protein, drug–indication, and phenotype–disease edges were downloaded from the NeDRexDB knowledgebase (54) (https://api.nedrex.net/). The STRING PPI network was obtained from STRING (https://string-db.org), and the ATC drug classification was retrieved from PubChem (https://pubchem.ncbi.nlm.nih.gov/classification/#hid=79). Due to licensing restrictions, we cannot redistribute the drug–protein interaction data derived from DrugBank. Researchers interested in accessing this data can apply for an academic license directly through DrugBank (https://www.drugbank.ca/).

## Abbreviations

ADR: Adverse drug reaction
DP: Disease phenotype
KG: Knowledge graph
SOC: System organ class
PT: Preferred term
LLT: Low level term
PPI: Protein–protein interaction

## Code availability

We provide open-source R and Python codes that allow the reader to replicate the results in this work and are available via the GitHub repository: https://github.com/faren-f/ADR_DP_similarity.
