# Supplementary material for: Phenotypic similarity of adverse drug reactions and disease phenotypes is a bridge to mechanistic discovery
Source: NPJ Drug Discov. 2025 Aug 4;2:19. doi: 10.1038/s44386-025-00021-6 (PMC13267064; doi:10.1038/s44386-025-00021-6)
Supplement: Supplementary file 1 — Supplementary Information [file 44386_2025_21_MOESM1_ESM.pdf]

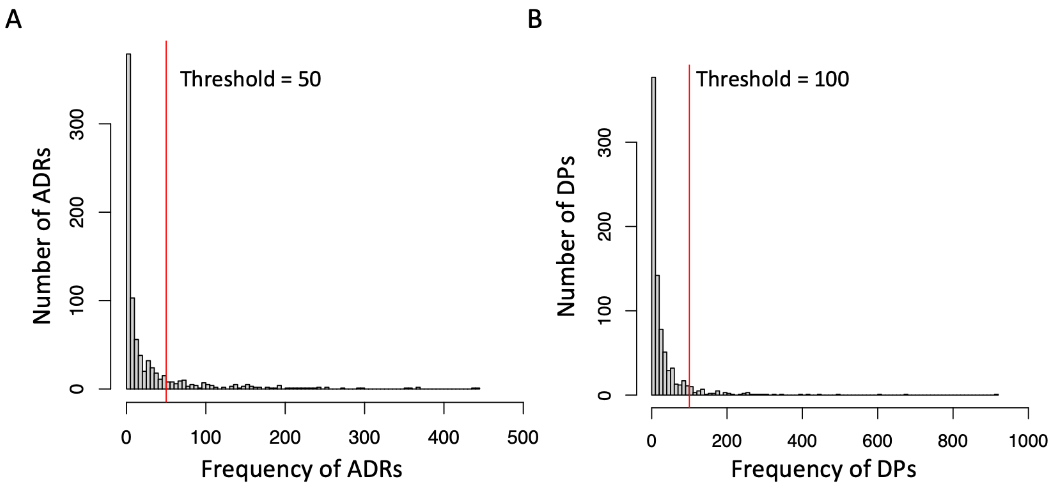

Fig. S1. Frequency distribution of adverse drug reactions (ADRs) and disease phenotypes (DPs) used for filtering common phenotypes. Histograms show the distribution of (A) ADR frequencies and (B) DP frequencies, measured by the number of drugs (for ADRs) or diseases (for DPs) associated with each phenotype. Red vertical lines indicate the thresholds applied to filter out overly frequent phenotypes (i.e., 50 for ADRs and 100 for DPs).

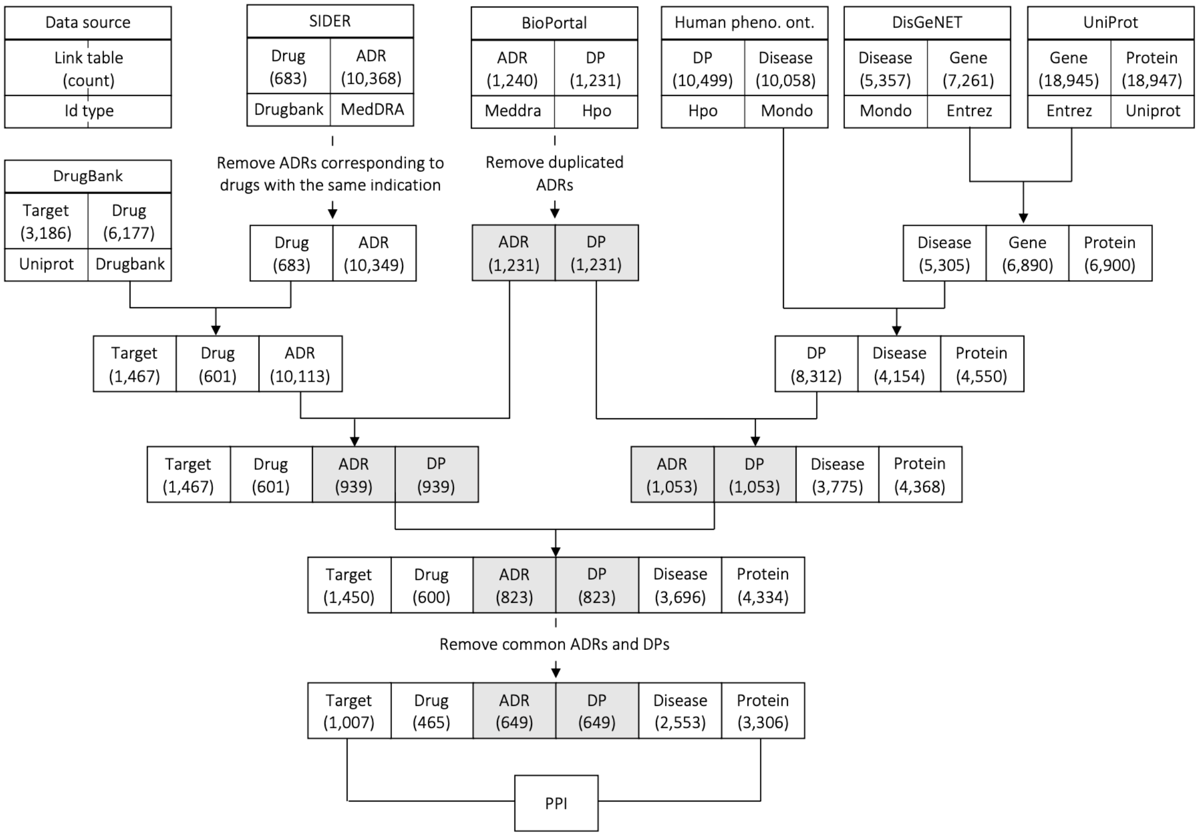

Fig. S2. Overview of knowledge graph construction, data sources, and pre-processing steps. The figure illustrates the entity types (e.g., drugs, diseases, proteins, ADRs, DPs), associated data sources (e.g., DrugBank, DisGeNET, MedDRA), and the steps applied for filtering, integration and refinement process to build our knowledge graph (adopted from (Firoozbakht et al. 2025))

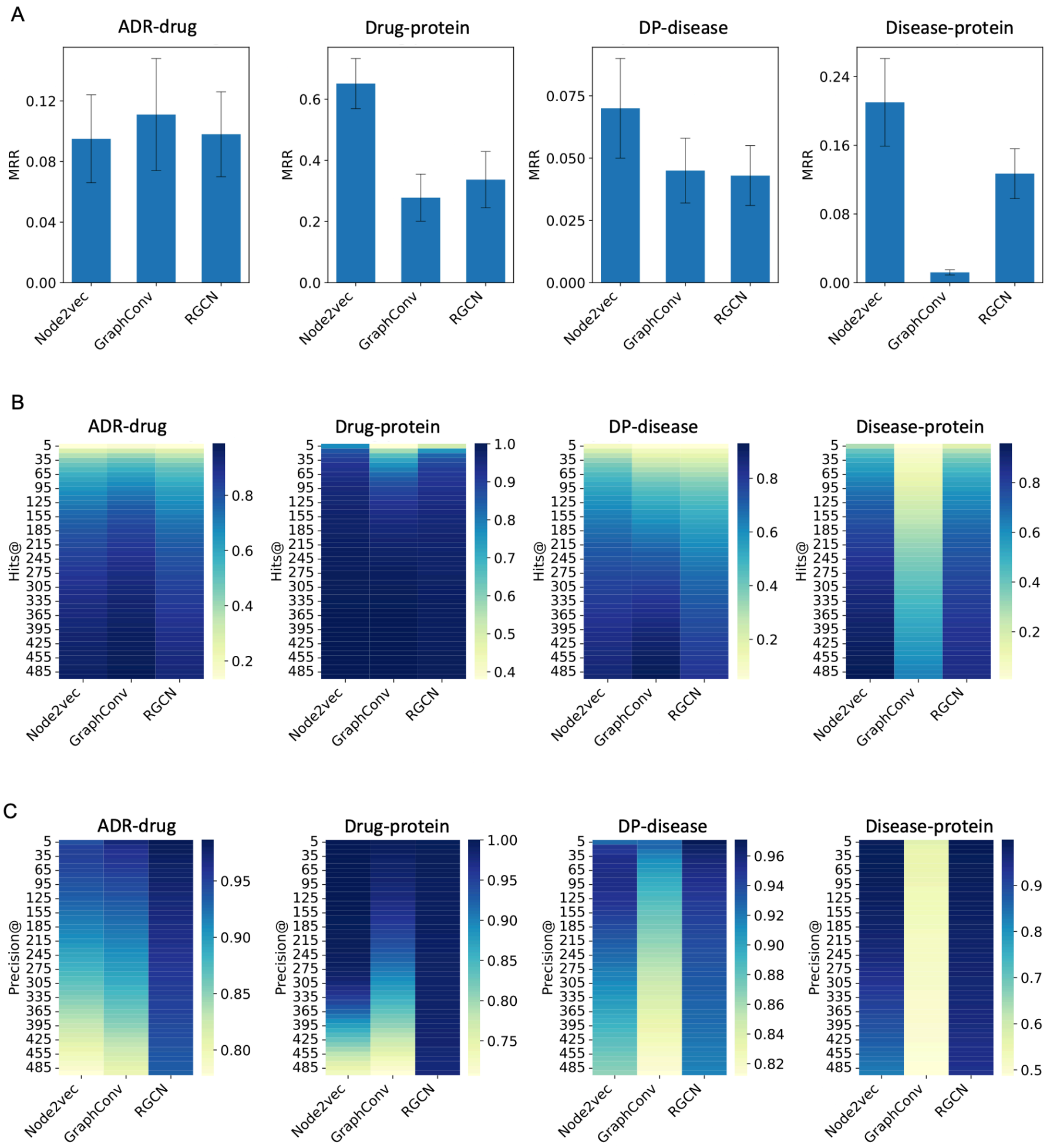

Fig. S3. Comparing link prediction results for different graph representation learning methods based on (**A**) mean reciprocal rank (MRR), (**B**) hits, and (**C**) precision.

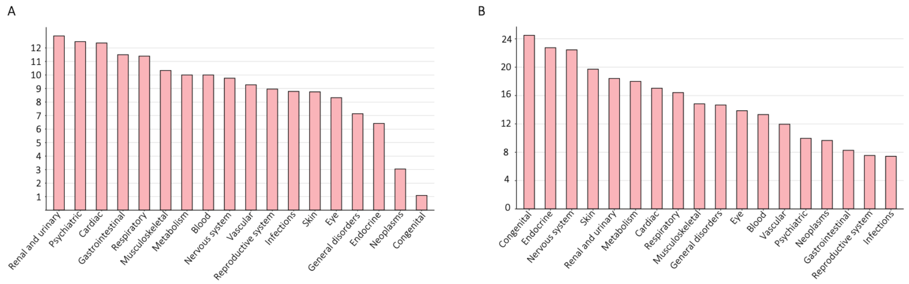

Fig. S4. (**A**) average number of drugs linked to ADRs in each SOC, (**B**) average number of diseases linked to DPs in each SOC.

Table S1. Link prediction results for different graph representation learning methods.

**Edge type**		**hit@5**	**precision@5**	**hit@ 50**	**precision@ 50**	**hit@ 100**	**precision @100**	**MRR**
**Drug-protein**	**Node2vec**	0.76	0.94	0.92	0.95	0.95	0.94	0.65
	**GraphConv**	0.38	0.97	0.79	0.95	0.89	0.94	0.27
	**RGCN**	0.50	0.98	0.89	0.97	0.93	0.97	0.33
	**Random**	0.00	0.20	0.09	0.41	0.18	0.36	0.00
**Drug-ADR**	**Node2vec**	0.12	1.0	0.53	1.0	0.67	0.99	0.09
	**GraphConv**	0.15	0.99	0.57	0.98	0.71	0.99	0.1
	**RGCN**	0.13	0.99	0.50	0.99	0.63	0.99	0.09
	**Random**	0.00	0.40	0.11	0.52	0.19	0.53	0.01
**Disease-DP**	**Node2vec**	0.12	0.93	0.41	0.95	0.54	0.95	0.07
	**GraphConv**	0.05	0.92	0.31	0.90	0.46	0.95	0.04
	**RGCN**	0.05	0.97	0.29	0.95	0.42	0.95	0.04
	**Random**	0.00	0.20	0.04	0.32	0.09	0.40	0.00
**Disease-protein**	**Node2vec**	0.27	0.99	0.56	0.99	0.66	0.99	0.2
	**GraphConv**	0.00	0.57	0.09	0.55	0.17	0.53	0.01
	**RGCN**	0.16	0.99	0.46	0.99	0.58	0.98	0.12
	**Random**	0.00	0.00	0.07	0.43	0.18	0.46	0.00
**Average**	**Node2vec**	**0.32**	0.96	**0.60**	**0.97**	**0.70**	**0.97**	**0.25**
	**GraphConv**	0.14	0.86	0.44	0.84	0.55	0.85	0.10

Edge type		hit@5	precision@5	hit@ 50	precision@ 50	hit@ 100	precision @100	MRR
**Drug-protein**	**Node2vec**	0.76	0.94	0.92	0.95	0.95	0.94	0.65
	**GraphConv**	0.38	0.97	0.79	0.95	0.89	0.94	0.27
	**RGCN**	0.21	**0.98**	0.53	**0.97**	0.64	**0.97**	0.14
	**Random**	0.00	0.2	0.07	0.42	0.16	0.44	0.00

Table S2. Enriched pathways for proteins with highest similarities to LLT-similar adverse drug reaction (ADR) and disease phenotype (DP) pairs. SOC: system organ class.

ADR	SOC	Enriched pathways
metabolic alkalosis	Metabolism	Cardiac conduction<br>Muscle contraction<br>Potassium Channels<br>Phase 0-rapid depolarisation<br>Phase 4 resting membrane potential
paroxysmal atrial fibrillation	Cardiac	Neuronal System<br>Muscle contraction<br>Cardiac conduction<br>Potassium Channels<br>Voltage gated Potassium channels
sleep paralysis	Nervous	GPCR ligand binding<br>Class A/1 (Rhodopsin−like receptors)<br>Peptide ligand−binding receptors<br>Class B/2 (Secretin family receptors)<br>Serotonin receptors
somnambulism	Psychiatric	GPCR ligand binding<br>Class A/1 (Rhodopsin−like receptors)<br>Transmission across Chemical Synapses<br>Amine ligand−binding receptors<br>Serotonin receptors
epidural hemorrhage	Injury	Formation of Fibrin Clot (Clotting Cascade)<br>Common Pathway of Fibrin Clot Formation<br>Intrinsic Pathway of Fibrin Clot Formation<br>Defects of contact activation system (CAS) and kallikrein/kinin system (KKS)<br>Diseases of hemostasis
shivering	General	Neuronal System<br>Transmission across Chemical Synapses<br>Neurotransmitter receptors and postsynaptic signal transmission<br>Amine ligand−binding receptors<br>Serotonin receptors

nocturia	Renal	Peptide ligand−binding receptors<br>Class A/1 (Rhodopsin−like receptors)<br>GPCR ligand binding<br>G alpha (q) signalling events

Table S3. Number of phenotypes in the original knowledge graph (KG), the reduced KG, and after excluding potential confounding drugs.

SOC	Number of phenotypes	Number of phenotypes of reduced KG	Number of phenotypes after confounder removal
Nervous	63	58	52
Psychiatric	28	26	16
Metabolism	38	37	33
Cardiac	30	29	26
Renal	27	25	22
Reproductive	24	24	17
Endocrine	12	12	12
Hepatobiliary	10	10	10
Musculoskeletal	30	28	24

Table S4. P-values representing the significance of embedding similarities for SOC-similar ADR-DPs across different system organ classes (SOCs) before and after excluding potential confounding drugs.

SOCs whose confounding drugs are excluded	P-values after excluding confounding drugs for each SOC								P-values before excluding confounding drugs
	Nervous	Psychiatric	Metabolism	Cardiac	Renal	Reproductive	Endocrine	Hepatobiliary	
Nervous	1.0	4e-01	1e-10	1.0	9e-01	3e-14	8e-01	1e-01	2e-95
Psychiatric	9e-04	1e-08	1e-136	1e-144	5e-16	8e-14	5e-06	3e-04	1e-144
Metabolism	6e-98	2e-106	2e-102	1e-123	8e-10	4e-22	2e-03	3e-06	1e-151
Cardiac	5e-111	9e-151	3e-180	5e-58	4e-25	2e-10	1e-04	1e-06	1e-131
Renal	1e-88	1e-123	2e-173	3e-124	1e-09	1e-08	3e-04	2e-05	1e-21
Reproductive	1e-66	2e-84	1e-138	1e-90	2e-19	2e-05	1e-03	3e-03	7e-08
Endocrine	3e-90	7e-126	5e-112	5e-139	4e-15	4e-05	8e-02	4e-04	7e-04

Hepatobiliary	1e-112	2e-154	1e-168	3e-121	4e-19	9e-07	1e-03	1e-05	3e-05

Table S5. Percentage of excluded drugs and drug–ADR links before and after excluding potential confounding drugs.

SOC	Percentage of excluded drugs from the reduced KG	Percentage of excluded ADR–drug links from the reduced KG
Nervous	28%	59%
Psychiatric	17%	52%
Metabolism	11%	25%
Cardiac	9%	10%
Renal	7%	13%
Reproductive	10%	22%
Endocrine	3%	10%
Hepatobiliary	2%	9%

Table S6. Adjusted p-values for the significant enrichment of pharmacological drug classes across SOCs.

MOA	Cardiac	Metabolism	Psychiatric	Renal	Reproductive	Hepatobiliary
A02-Drugs for acid related disorders	1.0	0.8	0.9	1.0	1.0	0.8
A03-Drugs for functional gastrointestinal disorders	1.0	–	–	1.0	1.0	0.8
A04-Antiemetics and antinauseants	1.0	–	–	–	–	–
A07-Antidiarrheals, intestinal anti inflammatory	1.0	1.0	0.8	1.0	1.0	0.8
A08-Antiobesity preparations, excl. diet products	–	–	0.8	1.0	1.0	0.8
A10-Drugs used in diabetes	–	–	–	1.0	–	0.8
A16-Other alimentary tract and metabolism products	–	–	–	1.0	1.0	–
B01-Antithrombotic agents	1.0	0.9	–	1.0	1.0	1.0
B03-Antianemic preparations	–	0.3	–	–	–	–
B05-Blood substitutes and perfusion solutions	–	0.8	–	–	–	–
C01-Cardiac therapy	1.0	0.8	0.8	1.0	–	0.9
C02-Antihypertensives	1.0	–	1.0	1.0	1.0	1.0

C03-Diuretics	–	0.8	–	1.0	–	0.8
C05-Vasoprotectives	1.0	1.0	–	1.0	–	–
C07-Beta blocking agents	0.8	0.8	0.8	–	–	0.8
C08-Calcium channel blockers	1.0	0.9	–	1.0	–	1.0
C09-Agents acting on the renin-angiotensin system	1.0	0.8	–	1.0	–	–
C10-Lipid modifying agents	–	0.9	–	1.0	–	0.8
D01-Antifungals for dermatological use	–	–	–	–	1.0	0.8
D04-Antipruritics, incl. antihistamines, anesthetics, etc.	1.0	0.8	0.8	1.0	–	0.8
D05-Antipsoriatics	–	1.0	0.8	1.0	–	–
D06-Antibiotics and chemotherapeutics for dermatological use	1.0	0.8	–	1.0	1.0	–
D10-Anti-acne preparations	–	1.0	–	–	–	–
G01-Gynecological antiinfectives and antiseptics	–	–	–	–	1.0	0.8
G02-Other gynecological	1.0	0.8	1.0	1.0	1.0	–
G03-Sex hormones and modulators of the genital system	1.0	1.0	1.0	1.0	0.3	1.0
G04-Urologicals	1.0	–	–	1.0	1.0	–
H01-Pituitary and hypothalamic hormones and analogues	1.0	1.0	–	–	1.0	0.8
H02-Corticosteroids for systemic use	–	–	–	–	1.0	0.8
J01-Antibacterials for systemic use	1.0	–	0.8	1.0	1.0	0.8
J02-Antimycotics for systemic use	1.0	0.8	–	1.0	1.0	0.8
J05-Antivirals for systemic use	–	–	0.8	–	–	–
L01-Antineoplastic agents	1.0	0.8	–	1.0	1.0	0.9
L02-Endocrine therapy	–	0.9	–	1.0	0.7	–
L04-Immunosuppressants	1.0	0.8	–	1.0	1.0	0.8
M01-Antiinflammatory and antirheumatic products	–	0.8	1.0	1.0	1.0	–
M02-Topical products for joint and muscular pain	–	0.9	1.0	1.0	1.0	–
M03-Muscle relaxants	1.0	1.0	0.8	1.0	1.0	0.8
M04-Antigout preparations	–	0.8	–	1.0	1.0	–
M05-Drugs for treatment of bone diseases	–	0.8	–	1.0	–	–
N01-Anesthetics	1.0	1.0	–	1.0	1.0	0.9
N02-Analgesics	1.0	0.8	0.5	1.0	1.0	0.8
N03-Antiepileptics	1.0	0.8	0.3	1.0	1.0	1.0
N04-Anti-parkinson drugs	1.0	0.8	0.5	1.0	1.0	0.8
N05-Psycholeptics	0.8	0.8	0.3	1.0	0.4	0.8

N06-Psychoanaleptics	0.5	0.8	**0.02**	1.0	1.0	0.8
N07-Other nervous system drugs	1.0	1.0	0.8	1.0	1.0	1.0
P01-Antiprotozoals	1.0	–	0.8	–	–	–
R02-Throat preparations	–	0.8	–	–	–	–
R03-Drugs for obstructive airway diseases	1.0	0.9	0.8	–	–	–
R06-Antihistamines for systemic use	–	–	1.0	1.0	–	1.0
S01-Ophthalmologica	1.0	0.8	0.8	1.0	1.0	0.8
V03-All other therapeutic products	–	0.9	–	–	–	–
V08-Contrast media	–	0.8	–	–	–	–
V09-Diagnostic radiopharmaceuticals	–	0.8	–	–	–	–

Reference

Firoozbakht, Farzaneh, Maria Louise Elkjaer, Diane E. Handy, Rui-Sheng Wang, Zoe Chervontseva, Matthias Rarey, Joseph Loscalzo, Jan Baumbach, and Olga Tsoy. 2025. "Exploring Common Mechanisms of Adverse Drug Reactions and Disease Phenotypes through Network-Based Analysis." *Cell Reports Methods* 5 (2): 100990.
